# Analytical study on childhood obesity and its association with parental lifestyle behaviors in family medicine clinics

**DOI:** 10.6026/973206300213039

**Published:** 2025-09-30

**Authors:** Akhshayaa Gunasekaran, Gayathri Kizhumuri Rajan, Ashok Nimmakanty Ramadas, Sushmitha Rameshbabu, Kiran Tawalare, Shobha Pajai, Mohamed Zaki Ullah Shareef

**Affiliations:** 1 Department of Paediatrics, Prince Charles Hospital, Cwm Taf Morgannwg University Health Board, Wales, United Kingdom; 2 Department of Paediatric Surgery, St. John's Medical College and Hospital, Bangalore, Karnataka, India; 3 Department of Pediatrics, General Physician, Madras Medical College, Chennai, Tamilnadu, India; 4 Department of Internal Medicine, Institute of Internal Medicine, Madras Medical College, Chennai, India; 5 Department of General Medicine, Shri Ayurved College, Nagpur, Maharashtra, India; 6 Department of Physiology, MGIMS, Sevagram, Maharashtra, India; 7 Department of Internal Medicine, AL Hada Armed Forces Hospital, TAIF, Kingdom of Saudi Arabia

**Keywords:** Childhood obesity, parental behavior, lifestyle, family medicine, physical activity, nutrition

## Abstract

The relationship between childhood obesity and parental lifestyle behaviors among children attending family medicine clinics. A total of 142 children aged 6-12 years and their parents were enrolled. Data on children's BMI and parents' physical activity, dietary habits, screen time practices and smoking status were collected via structured interviews and anthropometric measurements. Statistically significant associations were found between childhood obesity and parental inactivity, poor dietary patterns and excessive family screen time. Thus, we show the role of family-centered interventions in managing pediatric obesity.

## Background:

Childhood obesity has become a growing public health concern globally, with rising prevalence even in primary care settings [1]. Obesity in children is associated with an increased risk of hypertension, insulin resistance, musculoskeletal problems and psychosocial challenges [2]. One of the most important yet modifiable contributors to childhood obesity is the lifestyle behavior of parents [3]. Children tend to mimic the dietary, physical activity and screen-time habits of their primary caregivers, making the family environment a critical determinant of pediatric health [4]. In family medicine a clinic, where a holistic approaches to care is emphasized, evaluating the connection between parental lifestyle and child health outcomes is particularly relevant [5]. Existing literature suggests that sedentary behaviors, unhealthy eating patterns and smoking within the household can create an obesogenic environment for children [6]. However, there remains a gap in localized and clinic-based data to guide interventions. Therefore, it is of interest to assess the association between childhood obesity and parental lifestyle behaviors among children attending family medicine clinics.

## Materials and Methods:

This analytical cross-sectional study was conducted across three urban family medicine clinics over a 6-month period. A total of 142 children aged 6-12 years and their respective parents or primary caregivers were included using a systematic sampling approach. Inclusion criteria consisted of children presenting for routine check-ups, minor illnesses, or vaccinations, whose parents consented to participate. Children with endocrine disorders, chronic illnesses, or those on long-term medications affecting weight were excluded. Anthropometric measurements were taken for all children, including height and weight, to calculate Body Mass Index (BMI) percentiles according to WHO growth charts. Children with BMI ≥95th percentile were classified as obese. Parents were interviewed using a pre-validated structured questionnaire assessing lifestyle behaviors: physical activity frequency (≥150 minutes/week vs. less), dietary patterns (high-calorie, processed food intake frequency), screen time (≥2 hours/day) and smoking status. Additional variables such as parental education, family income and household meal routines were also documented. Data was analyzed using SPSS software. Chi-square tests and independent t-tests were applied for categorical and continuous variables, respectively. Logistic regression was performed to identify independent predictors of childhood obesity. A p-value <0.05 was considered statistically significant.

## Results:

Among 142 children studied, 46 (32.4%) were classified as obese. Obesity was significantly associated with several parental lifestyle factors, including physical inactivity, high screen time practices and unhealthy dietary habits and smoking status. Multivariate logistic regression identified parental physical inactivity and high-calorie dietary patterns as independent predictors of childhood obesity, even after adjusting for socioeconomic variables. Table 1 (see PDF) shows Children of physically inactive parents showed significantly higher rates of obesity. Table 2 (see PDF) shows High family screen time was strongly correlated with child obesity. Table 3 (see PDF) shows Frequent consumption of processed and high-calorie foods by the family was significantly higher in the obese group. Table 4 (see PDF) shows Parental smoking showed a moderate association with obesity in children. Table 5 (see PDF) shows Obese children were more likely to come from households with irregular mealtimes. Table 6 (see PDF) shows No significant difference was found in mean parental education levels between groups. Table 7 (see PDF) shows higher monthly household income was associated with greater obesity prevalence in this cohort. Table 8 (see PDF) shows Obese children engaged significantly less in outdoor play compared to non-obese peers. Table 9 (see PDF) shows Use of devices during meals was higher among families with obese children. Table 10 (see PDF) shows Children from families that ate meals together regularly had lower obesity prevalence.

## Discussion:

This analytical study highlights a strong association between childhood obesity and specific parental lifestyle behaviors in a family medicine clinic setting. Children whose parents exhibited low levels of physical activity, frequently consumed unhealthy diets, permitted high screen time and smoked were significantly more likely to be obese [7]. These findings are consistent with the well-established concept that parental modeling plays a central role in shaping children's long-term health behaviors and weight outcomes [8]. Among all factors, parental physical inactivity and poor dietary habits emerged as the most significant independent predictors of childhood obesity. These findings reinforce prior research indicating that the home environment - particularly when it lacks structure around physical activity and healthy eating - fosters behaviors that contribute to excess weight gain in children [9, 10]. Notably, even after adjusting for socioeconomic status and education, lifestyle behaviors retained a stronger association with childhood obesity, suggesting that behavioral patterns rather than just income or education level are the more modifiable drivers. The study also found that family routines like irregular meal timings and frequent use of digital devices during meals correlated with higher obesity rates [11, 12]. Such disruptions may lead to mindless eating and reduced satiety cues, supporting literature that links structured family meals with better nutritional intake and weight control [13]. The association between parent who is overweight and their child who is overweight could be driven by parent's background or behaviors. Studies have found that parental educational status, occupational status, and parenting styles and feeding practices are some of the contributing factors to childhood obesity [14]. Underweight parents have healthier behaviors related obesity than obese parents [15]. Interestingly, children of smokers were also more likely to be obese, hinting at the possible clustering of multiple health-risk behaviors within households. While the data are cross-sectional and causality cannot be established, these findings carry clinical implications for primary care. Family physicians should not only monitor children's growth parameters but also engage parents in lifestyle counseling, as modifying adult behaviors may indirectly benefit pediatric outcomes [16, 17]. Future interventional studies should examine the long-term impact of targeted parental behavior modification on childhood weight trajectories.

## Conclusion:

Childhood obesity is strongly influenced by parental lifestyle behaviors, including physical inactivity, poor dietary habits and high screen time. Targeted family-based interventions in primary care settings can play a crucial role in obesity prevention. Encouraging structured routines and healthier habits at the family level is essential for long-term pediatric health.
